# Supplementary material for: Crop, Host, and Gut Microbiome Variation Influence Precision Nutrition: An Example of Blueberries
Source: Antioxidants (Basel). 2023 May 22;12(5):1136. doi: 10.3390/antiox12051136 (PMC10215964; doi:10.3390/antiox12051136)
Supplement: Supplementary file 1 [file antioxidants-12-01136-s001.zip › antioxidants-2382922-supplementary.pdf]

Supplementary Table S1. Pharmacokinetic Parameters for Anthocyanin Metabolites

Metabolite	Blueberry Genotype	AUC (μM*h)	Cmax (nM)	Tmax (h)	Normalized by dose	
					AUC (μM*h/mg)	Cmax (nM/mg)
Cy-3-Glc	Ira	0.02 ± 0	8.38 ± 1.53	0.33 ± 0.08	0.022 ± 0.005[c]	7.85 ± 1.43[c]
	Montgomery	0.05 ± 0.01	9.4 ± 1.12	0.42 ± 0.08	0.125 ± 0.018[a]	24.96 ± 3.72[a]
	Onslow	0.03 ± 0	10.4 ± 0.68	0.5 ± 0	0.041 ± 0.003[bc]	13.25 ± 0.76[bc]
	SHF2B1	0.04 ± 0.01	8.51 ± 1.14	0.67 ± 0.17	0.047 ± 0.008[bc]	10.82 ± 1.21[bc]
	LB composite	0.03 ± 0.01	10.18 ± 1.16	0.5 ± 0	0.054 ± 0.014[b]	17.38 ± 2.16[ab]
Del-3-Glc	Ira	0.03 ± 0.01	5.81 ± 0.56	0.5 ± 0.18	0.039 ± 0.012[ab]	8.25 ± 0.8[ab]
	Montgomery	0.03 ± 0.01	5.52 ± 1.16	0.67 ± 0.17	0.084 ± 0.029[a]	14.84 ± 3.55[a]
	Onslow	0.02 ± 0	4.71 ± 0.47	0.5 ± 0	0.021 ± 0.001[b]	6 ± 0.56[b]
	SHF2B1	0.04 ± 0.01	7.12 ± 0.91	0.69 ± 0.19	0.022 ± 0.001[b]	4.33 ± 0.59[b]
	LB composite	0.02 ± 0	4.6 ± 0	0.5 ± 0	0.024 ± 0.008[ab]	4.98 ± 0[b]
Mal-3-Glc	Ira	0.033 ± 0.01	7.99 ± 1.03	0.56 ± 0.16	0.027 ± 0.006[b]	6.42 ± 0.81[b]
	Montgomery	0.04 ± 0.01	10.8 ± 2.29	1 ± 0.35	0.076 ± 0.009[a]	14.65 ± 3.16[a]
	Onslow	0.03 ± 0.01	10.07 ± 0.56	0.5 ± 0	0.028 ± 0.004[b]	8.83 ± 0.4[ab]
	SHF2B1	0.04 ± 0.01	10.05 ± 2.05	0.56 ± 0.16	0.022 ± 0.003[b]	5.53 ± 1.04[b]
	LB composite	0.04 ± 0.01	11.51 ± 2.23	0.5 ± 0	0.026 ± 0.006[b]	6.71 ± 1.38[b]
Peo-3-Glc	Ira	0.03 ± 0.01	6.36 ± 0.98	0.44 ± 0.06	0.035 ± 0.014	8.14 ± 1.25[c]
	Montgomery	0.03 ± 0.01	7.87 ± 0.96	0.56 ± 0.16	0.106 ± 0.037	28.99 ± 3.94[a]
	Onslow	0.02 ± 0	8.18 ± 0.46	30 ± 0	0.028 ± 0.006	10.93 ± 0.49[bc]
	SHF2B1	0.04 ± 0.01	8.75 ± 1.45	0.69 ± 0.19	0.088 ± 0.024	19.11 ± 2.81[abc]
	LB composite	0.02 ± 0.01	11.25 ± 1.46	0.38 ± 0.13	0.055 ± 0.007	20.39 ± 2.91[ab]
Pet-3-Glc	Ira	0.03 ± 0.01	6.44 ± 0.79	0.44 ± 0.06	0.042 ± 0.014	10.34 ± 1.26[ab]
	Montgomery	0.03 ± 0.01	7.2 ± 1.81	0.56 ± 0.16	0.093 ± 0.042	25.98 ± 6.71[a]
	Onslow	0.03 ± 0	8.37 ± 0.53	0.5 ± 0	0.044 ± 0.006	13.08 ± 0.71[ab]
	SHF2B1	0.04 ± 0.01	8.26 ± 1.32	0.69 ± 0.19	0.029 ± 0.007	6.6 ± 0.93[b]
	LB composite	0.03 ± 0.01	8.89 ± 1.74	0.88 ± 0.38	0.038 ± 0.009	12.42 ± 2.58[ab]

Data represented as mean ± SEM (n=4 rats / group). Different letters represent significant differences between blueberry genotypes, within each metabolite ($p < 0.05$). Data represented as mean ± SEM (n = 3-4 rats / group). Cy-3-Glcs, cyanidin-3-glucosides; Del-3-Glcs, delphinidin-3-glucosides; Mal-3-Glcs, malvidin-3-glucosides; Peo-3-Glcs, peonidin-3-glucosides; Pet-3-Glcs, petunidin-3-glucosides.

Supplementary Table S2. Pharmacokinetic Parameters for Flavan-3-ol Metabolites

Metabolite	Genotype	AUC (µmol/L*h)	Cmax (µM)	Tmax (h)	AUC (µM*h/mg)	Cmax (µM/mg)
					Normalized by Dose	
C-Glcr	Ira	0.098 ± 0.016	0.040 ± 0.006[a]	1.125 ± 0.315	2.386 ± 0.378	0.984 ± 0.154
	Montgomery	0.089 ± 0.013	0.034 ± 0.005[ab]	1.375 ± 0.375	2.454 ± 0.401	0.932 ± 0.145
	Onslow	0.099 ± 0.025	0.037 ± 0.007[ab]	1.250 ± 0.250	2.519 ± 0.619	0.955 ± 0.163
	SHF2B1-21:3	0.051 ± 0.016	0.020 ± 0.004[ab]	0.500 ± 0.000	2.572 ± 0.775	1.036 ± 0.199
	LB composite	0.037 ± 0.016	0.015 ± 0.005[b]	1.875 ± 0.774	2.456 ± 1.054	1.031 ± 0.362
MeC-Glcr	Ira	0.154 ± 0.016	0.039 ± 0.005	1.125 ± 0.315	3.764 ± 0.380	0.962 ± 0.120
	Montgomery	0.134 ± 0.017	0.042 ± 0.007	1.500 ± 0.289	3.658 ± 0.515	1.078 ± 0.157
	Onslow	0.136 ± 0.036	0.039 ± 0.008	1.125 ± 0.315	3.442 ± 0.880	1.076 ± 0.172
	SHF2B1-21:3	0.096 ± 0.030	0.024 ± 0.005	0.750 ± 0.144	4.814 ± 1.425	2.000 ± 0.365
	LB composite	0.089 ± 0.033	0.021 ± 0.007	1.875 ± 0.774	5.874 ± 2.213	1.623 ± 0.359
EC-Glcr	Ira	0.027 ± 0.005	0.013 ± 0.002	1.000 ± 0.354	9.896 ± 1.734	4.631 ± 0.880
	Montgomery	0.044 ± 0.005	0.018 ± 0.003	1.375 ± 0.375	9.448 ± 1.268	3.799 ± 0.701
	Onslow	0.065 ± 0.017	0.024 ± 0.004	1.500 ± 0.289	7.793 ± 2.010	2.855 ± 0.429
	SHF2B1-21:3	0.048 ± 0.018	0.017 ± 0.004	0.750 ± 0.144	8.942 ± 3.085	3.079 ± 0.743
	LB composite	0.033 ± 0.011	0.014 ± 0.003	1.375 ± 0.875	10.358 ± 3.559	4.533 ± 1.634
MeEC-Glcr	Ira	0.045 ± 0.011	0.013 ± 0.002	0.750 ± 0.144	0.016 ± 0.004	0.007 ± 0.003
	Montgomery	0.054 ± 0.014	0.018 ± 0.003	1.250 ± 0.250	0.011 ± 0.003	0.003 ± 0.001
	Onslow	0.071 ± 0.023	0.024 ± 0.004	1.250 ± 0.250	0.008 ± 0.003	0.002 ± 0.001

	SHF2B1-21:3	0.061	±	0.022	0.017	±	0.004	1.125	±	0.315	0.011	±	0.004	0.004	±	0.001
	LB composite	0.051	±	0.020	0.014	±	0.003	1.500	±	0.289	0.016	±	0.006	0.005	±	0.001

Data represented as mean ± SEM (n=4 rats / group). Letters represent significant differences ($p < 0.05$) in metabolites between genotypes. EC-glcr, epicatechin-5-glucuronide; C-glcr, catechin-5-glucuronide; EC-gclr, 3'-O-methylepicatechin-5-glcr; MeC-glcr, 3'-O-methylcatechin-5-glcr.

Supplementary Table S3. Pharmacokinetic parameters for flavan-3-ol metabolites

Metabolite	Genotype	AUC (µmol/L*h)	Cmax (µM)	Tmax (h)	AUC (µM*h/mg)	Cmax (µM/mg)
					Normalized by Dose	
Q-Gclr	Ira	0.200 ± 0.029	0.096 ± 0.015	0.500 ± 0.000	0.718 ± 0.098^{ab}	0.046 ± 0.018^{d}
	Montgomery	0.312 ± 0.082	0.173 ± 0.038	0.625 ± 0.125	1.191 ± 0.342^{a}	0.658 ± 0.160^{a}
	Onslow	0.258 ± 0.027	0.135 ± 0.022	0.500 ± 0.000	0.763 ± 0.071^{ab}	0.402 ± 0.066^{ab}
	SHF2B1-21:3	0.193 ± 0.026	0.116 ± 0.013	0.500 ± 0.000	0.355 ± 0.050^{b}	0.215 ± 0.026^{bc}
	LB composite	0.334 ± 0.040	0.111 ± 0.038	1.000 ± 0.354	0.424 ± 0.051^{b}	0.141 ± 0.023^{c}
MeQ-Gclr	Ira	0.026 ± 0.026	0.005 ± 0.005	2.000 ± 0.000	0.093 ± 0.093^{c}	0.004 ± 0.004^{c}
	Montgomery	0.185 ± 0.053	0.074 ± 0.017	1.250 ± 0.433	0.705 ± 0.218^{a}	0.281 ± 0.070^{a}
	Onslow	0.146 ± 0.035	0.059 ± 0.007	0.875 ± 0.375	0.432 ± 0.100^{ab}	0.174 ± 0.021^{ab}
	SHF2B1-21:3	0.132 ± 0.034	0.049 ± 0.008	0.500 ± 0.000	0.242 ± 0.060^{ab}	0.090 ± 0.015^{b}
	LB composite	0.242 ± 0.054	0.056 ± 0.008	1.875 ± 0.774	0.309 ± 0.073^{ab}	0.071 ± 0.011^{b}
Myr-Gclr	Ira	1.779 ± 0.201^{a}	0.492 ± 0.024^{a}	0.875 ± 0.375^{c}	0.315 ± 0.038^{a}	0.087 ± 0.005^{a}
	Montgomery	2.345 ± 0.344^{a}	0.768 ± 0.132^{a}	2.000 ± 0.000^{ab}	0.168 ± 0.028^{b}	0.055 ± 0.011^{b}
	Onslow	0.696 ± 0.028^{b}	0.226 ± 0.010^{b}	1.250 ± 0.250^{bc}	0.115 ± 0.005^{b}	0.037 ± 0.002^{b}
	SHF2B1-21:3	0.219 ± 0.022^{c}	0.068 ± 0.004^{c}	0.875 ± 0.125^{c}	0.001 ± 0.000^{c}	0.000 ± 0.000^{c}
	LB composite	0.746 ± 0.111^{b}	0.195 ± 0.020^{b}	3.250 ± 0.750^{a}	0.014 ± 0.002^{c}	0.004 ± 0.000^{c}

Letters represent significant differences ($p < 0.05$) in pharmacokinetic parameters between blueberry genotypes for each metabolite. Q-Gclr, quercetin-3-glucuronide; MeQ-Gclr, methylquercetin-3-glucuronide; Myr-Gclr, myricetin glucuronide. Data represented as mean ± SEM (n=4 rats / group).

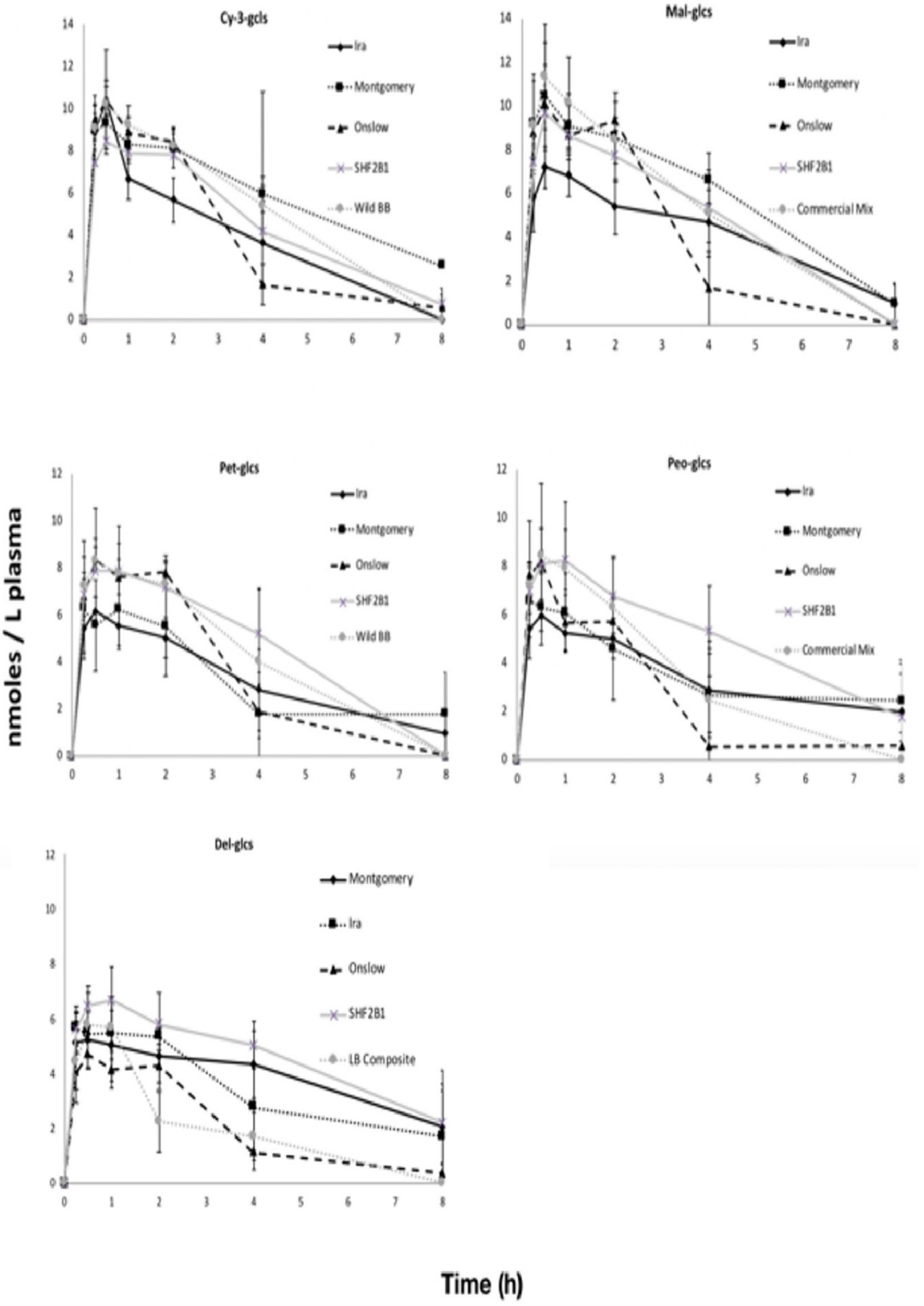

Supplementary Figure S1. Plasma pharmacokinetic response of anthocyanin metabolites from different blueberry genotypes. Data represented as mean ± SEM (n=4 rats / group).

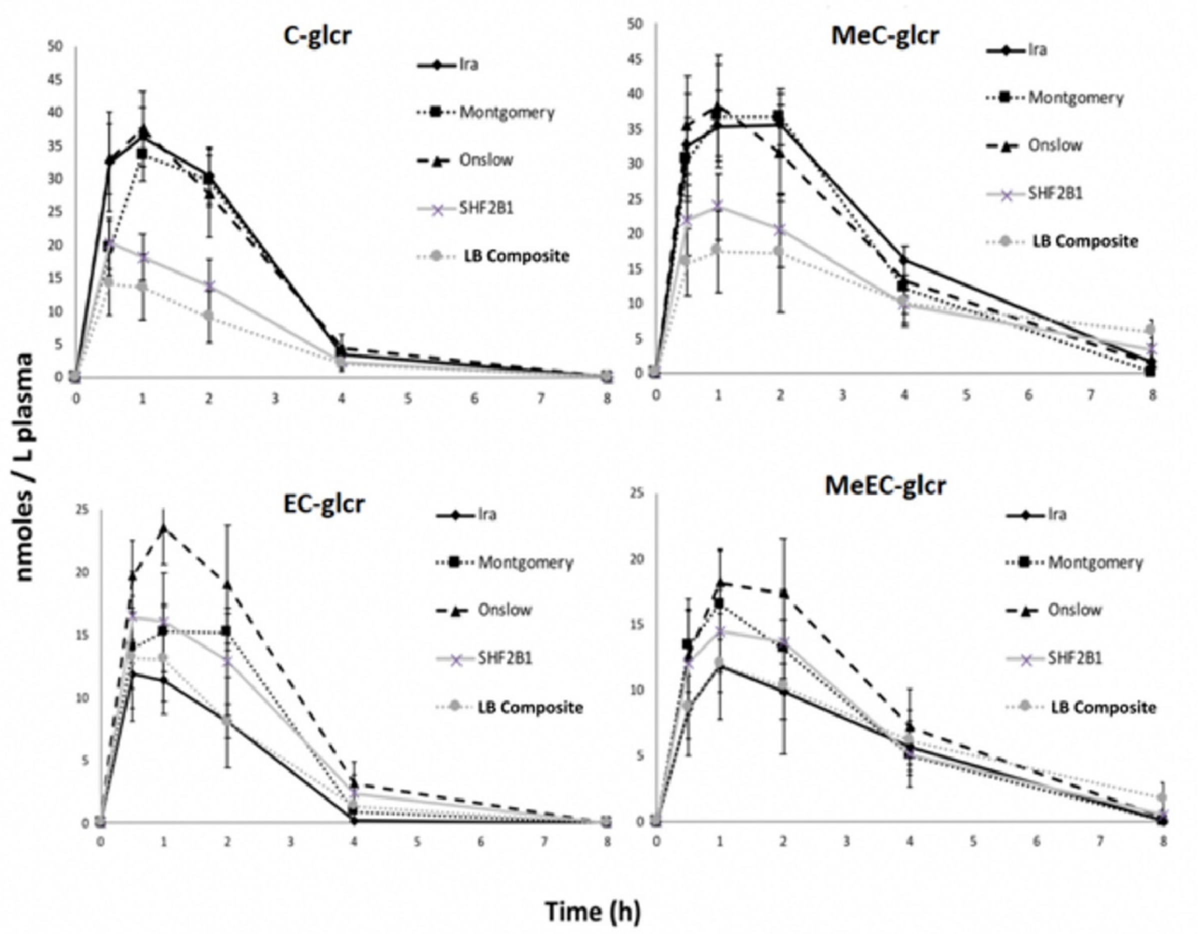

Supplementary Figure S2. Plasma pharmacokinetic response of flavan-3-ols metabolites from different blueberry genotypes. Data represented as mean ± SEM (n = 4 rats / group). EC-glcr, epicatechin-5-glucuronide; C-glcr, catechin-5-glucuronide; EC-gclr, 3'-O-methylepicatechin-5-glcr; MeC-glcr, 3'-O-methylcatechin-5-glcr.

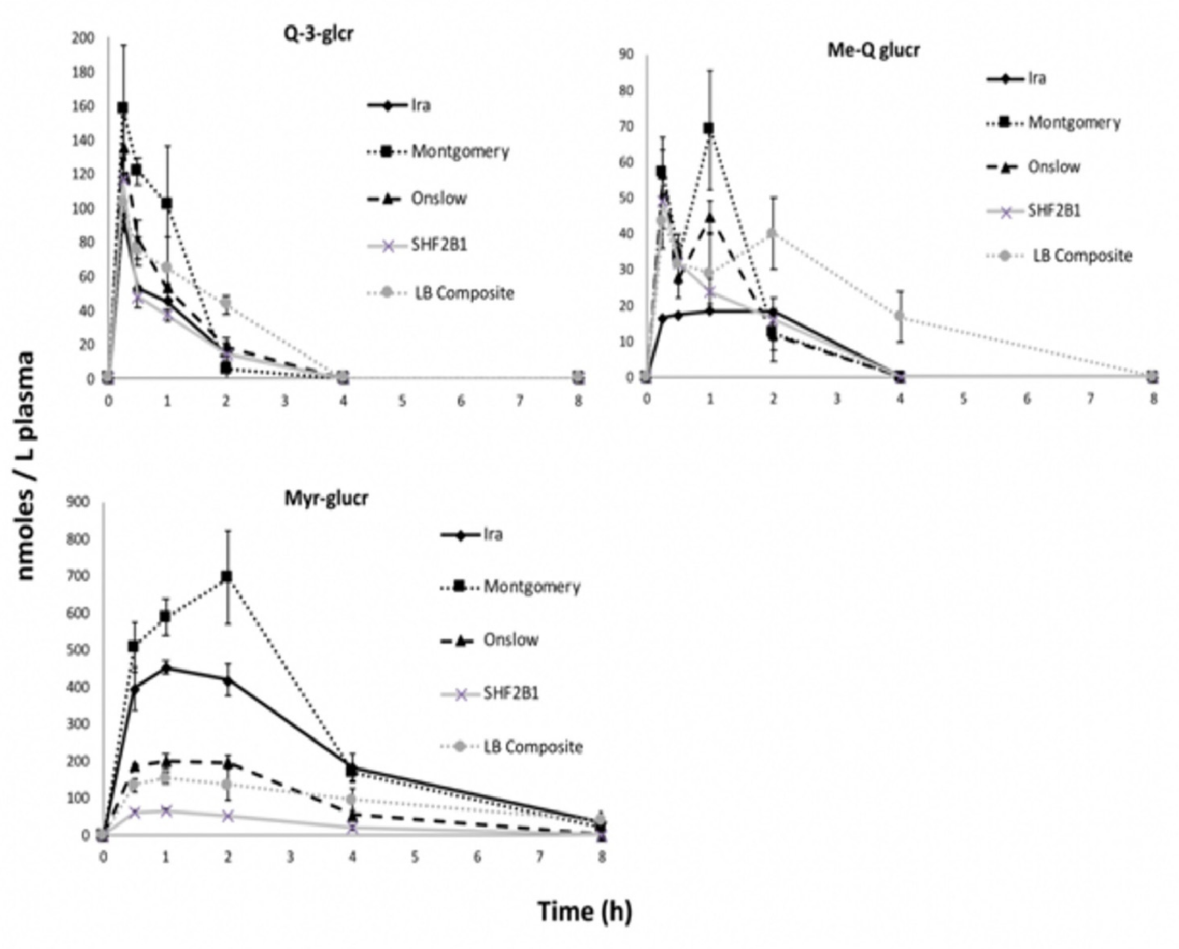

Supplementary Figure S3. Plasma pharmacokinetic response of flavonol metabolites from different blueberry genotypes. Data represented as mean ± SEM (n = 4 rats / group). Q-3-glcr, quercetin-3-glucuronide; Me-Q-glucr, methylquercetin-3-glucuronide; Myr-glucr, myricetin glucuronide.

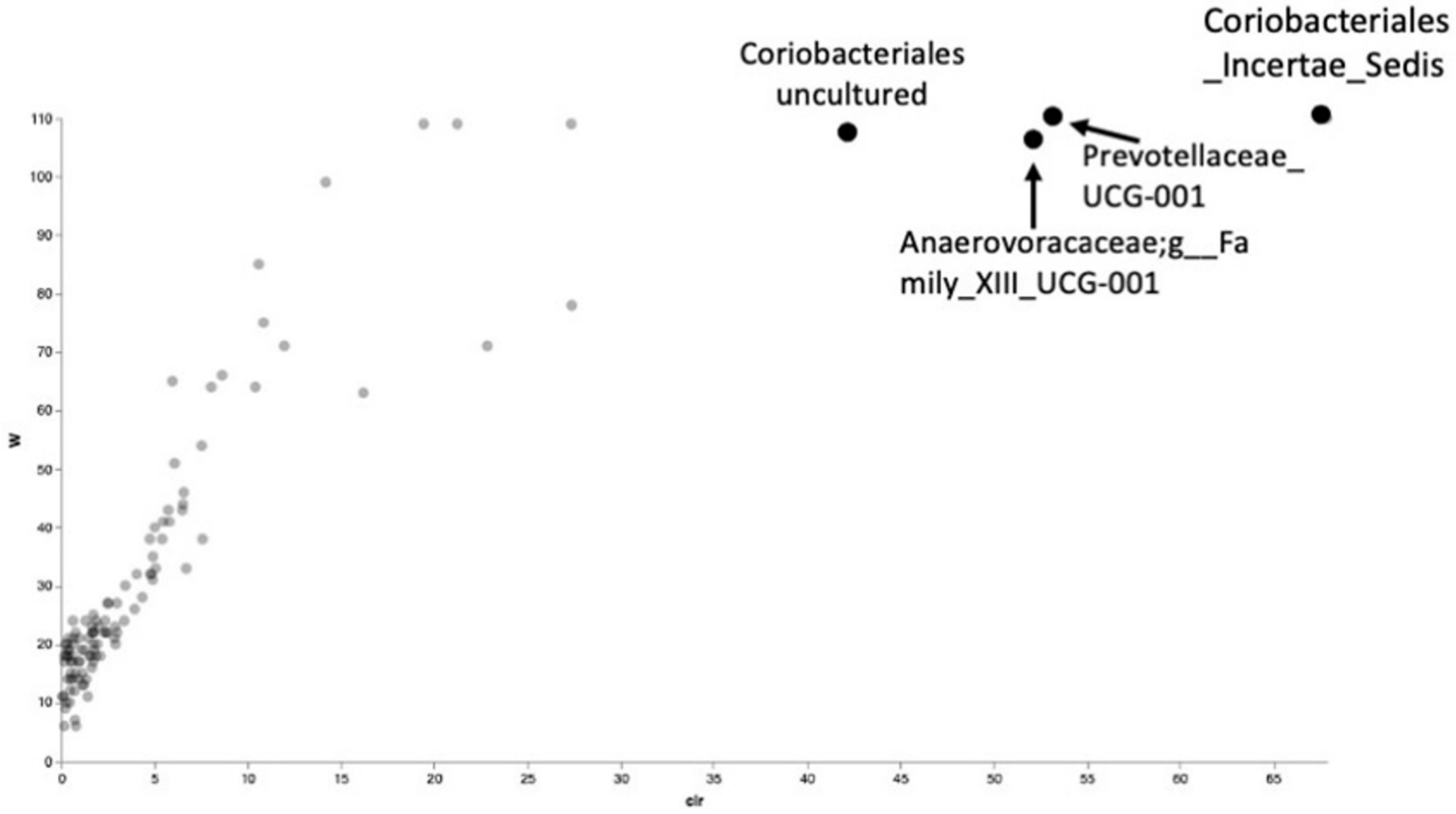

Figure S4. Volcano plots of taxa identified by Analysis of Composition of Microbiomes (ANCOM) as differentially abundant in different doses of blueberry diet. Taxa in the upper right side (clr >40 and W >100) of the figure are illustrated using box-plots in Figure 7. X-axis represented by center log ratio (clr) and y-axis is W is number of time null hypothesis was rejected.
